# Extremely high strength and work hardening ability in a metastable high entropy alloy

**DOI:** 10.1038/s41598-018-28383-0

**Published:** 2018-07-02

**Authors:** S. S. Nene, M. Frank, K. Liu, R. S. Mishra, B. A. McWilliams, K. C. Cho

**Affiliations:** 1 Center for Friction Stir Processing, Department of Materials Science and Engineering, University of North Texas, Denton, Texas 76207 USA; 2 Weapons and Materials Research Directorate, U.S. Army Research Laboratory, Aberdeen Proving Grounds, MD 21005 USA

## Abstract

Design of multi-phase high entropy alloys uses metastability of phases to tune the strain accommodation by favoring transformation and/or twinning during deformation. Inspired by this, here we present Si containing dual phase Fe_42_Mn_28_Co_10_Cr_15_Si_5_ high entropy alloy (DP-5Si-HEA) exhibiting very high strength (1.15 GPa) and work hardening (WH) ability. The addition of Si in DP-5Si-HEA decreased the stability of f.c.c. (γ) matrix thereby promoting pronounced transformation induced plastic deformation in both as-cast and grain refined DP-5Si-HEAs. Higher yet sustained WH ability in fine grained DP-5Si-HEA is associated with the uniform strain partitioning among the metastable γ phase and resultant h.c.p. (ε) phase thereby resulting in total elongation of 12%. Hence, design of dual phase HEAs for improved strength and work hardenability can be attained by tuning the metastability of γ matrix through proper choice of alloy chemistry from the abundant compositional space of HEAs.

## Introduction

The concepts of high entropy alloy (HEA) and transformation induced plasticity (TRIP) was recently merged by Li *et al*.^1,2^ in a dual phase Fe_50_Mn_30_Co_10_Cr_10_ high entropy alloy (DP-HEA). A major discovery in this work was the observation of concurrent increase in strength and ductility with refinement of grain size. The fundamental basis for this was TRIP in this DP-HEA and appropriately, Li *et al*.^1,2^ called it DP-TRIP-HEA. While the overall results were very exciting, the yield strength (YS) of the DP-TRIP-HEA was quite low, ~200–300 MPa range in as-homogenized condition and ~250–375 MPa after thermomechanical processing. They further tried to enhance the YS by addition of interstitial carbon and called it as DP-iHEA^3^. The C addition could not increase the YS significantly, however, reduced the tripping tendency of the material^3^. Moreover, our recent work showed that friction stir processing (FSP) of DP-HEA increased the YS of the material by 100 MPa while retaining the other mechanical properties reported by Li *et al*.^1,2^. These results suggested that, either the change in alloy chemistry or the processing path could result in enhanced YS but may lose the metastability driven TRIP or twinning induced plasticity (TWIP) effects in DP-HEAs. However, TRIP literature^2–7^ suggested that (h.c.p.) ε phase is harder than the γ matrix and hence evolution of martensite dominant microstructure would result in increased YS of the alloy while retaining the TRIP or TWIP effect. In view of this, martensite fraction in the microstructure can be increased by increasing the metastability of the (f.c.c.) γ phase. Moreover, recent results on TRIP assisted HEAs^1–4^ showed that presence of both Fe and Mn is needed for altering the γ phase stability during deformation and hence should be an integral part of the alloy chemistry. Along with transition elements, it is shown that, light weight elements like Si, C, Al have a massive effect on γ phase evolution in TRIP assisted Fe-Mn alloys^5–7^.

Thus, present study involved design of a TRIP assisted HEA with enhanced strength using two strands of motivation.

1. *Design of dual phase HEA with increased metastability of the γ matrix*: This altered phase stability in the HEA can be achieved by changing the alloy chemistry. The selection of the alloy chemistry was based on the literature available for TRIP assisted HEAs and steels along with the use of thermodynamic simulations by Thermo-Calc software.
2. *Microstructure engineering*: Microstructure of the newly designed HEAs was tailored using FSP. FSP being a unique high temperature severe deformation process that alters grain size and phase evolution. At the same time, shear driven transport of elements during FSP helps to retain the chemical homogeneity of the microstructure^4,8^.

## Alloy Design Approach

**Figure 1:**
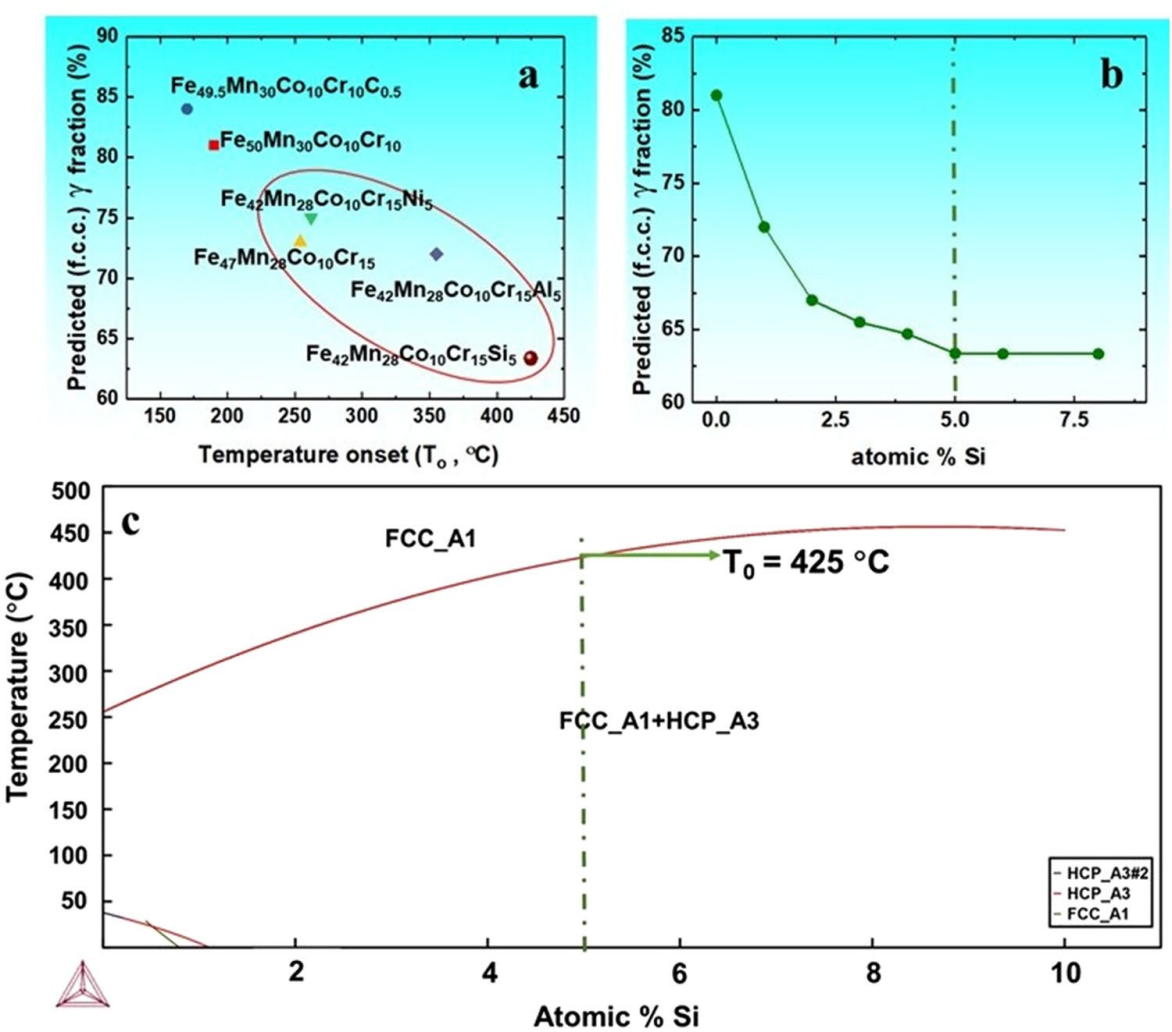
Thermodynamic phase predictions for metastable DP-HEA design using Thermo-Calc software. (**a**) Estimated equilibrium γ phase fractions and temperature onset (T_o_) for Fe_47−x_Mn_28_Co_10_Cr_15_Y_x_ (Y: Si or Al or Ni and x: at.% contribution of selected alloying element) system in comparison with Fe_50_Mn_30_Co_10_Cr_10_ and Fe_49.5_Mn_30_Co_10_Cr_10_C_0.5_ alloys; (**b**) Effect of Si content on equilibrium γ phase fraction using Thermo-Calc and; (**c**) Fe_47−x_Mn_28_Co_10_Cr_15_Si_x_ phase diagram obtained from Thermo-Calc showing increase in the T_o_ temperature with Si content. (Thermo-Calc simulations are based on high entropy alloy database TCHEA2).

The γ → ε transformation in the TRIP alloys is governed by the Gibb’s free energy for martensite formation (ΔG^γ→ε^) from the metastable γ phase. Therefore higher the metastability of the γ phase, higher is the driving force for TRIP to occur and lower is the value of ΔG^γ→ε^. Further, lower ΔG^γ→ε^ values also indicate a low stacking fault energy (SFE) of the system since ε formation (TRIP effect) is based on the pre-exixtance of stable intrincsic stacking faults in the microstructure. As a result, increasing γ metstability essentially corresponds to decrease in SFE of the system^8–11^. In other words, alloys with metastable γ matrix would result in lower equilibrium γ fraction at room temperature (25 °C) owing to higher driving force for transformation. Thus, room temperature equilibrium γ fraction obtained from Thermo-Calc simulations for a given alloy chemistry can also be considered as a measure for tuning the γ phase stability in the alloy design approach^5,8–11^. In line of this, thermodynamics simulations were made with Thermo-Calc software (HEA database; TCHEA2) to predict equilibrium γ fractions (at 25 °C) and temperature onset (temperature below which mixture of γ and ε phases exists) for Fe-Mn-Co-Cr containing HEAs (Fig. 1a–c) using the property diagrams. These simulations depict (Fig. 1a) very high equilibrium γ phase fractions and low temperature onset (T_o_) values for recently designed DP-HEA and DP-iHEA, respectively which indirectly demonstrate low γ phase metastability in them.

The earlier simulation work by Xiong *et al*.^5^ and Nava *et al*.^9^ showed that Cr increases the driving force for γ → ε transformation whereas Mn decreases it. Thus the equilibrium γ fraction and T_o_ values were estimated for the Fe_50−x_Mn_30−y_Co_10_Cr_10+x+y_ wherein Cr is increased at the expense of Fe and Mn content in Fe_50_Mn_30_Co_10_Cr_10_ system. Among that, Fe_47_Mn_28_Co_10_Cr_15_ (wherein x = 3, y = 2) alloy containing almost 5 at.% higher Cr compared to DP-HEA displayed decrease in predicted equilibrium γ phase fraction, however with marginal change in T_o_ value (yellow triangle in Fig. 1a). Further, extensive work by Xiong *et al*.^5^ on Fe-Mn alloys estimated the effect of non-transition alloying elements (i.e. Al, C, Si) on ΔG^γ→ε^ by thermodynamic simulations for given Mn content in the alloy. According to their work, C and Al increased the ΔG^γ→ε^ values (more γ phase fraction) whereas Si decreased it (less γ phase fraction).

As a result, effect of Si on the γ phase stability was studied by obtaining equilibrium γ phase fractions (300 K) for Fe_47−x_Mn_28_Co_10_Cr_15_Si_x_ (where x = 0 to 8 at.% Si) HEA (Fig. 1b) using Thermo-Calc simulations. Figure 1b shows the dramatic decrease in the γ fraction with increased Si content till 5 at.% beyond which the variation become sluggish. Moreover, the phase diagram (Fig. 1c) estimation for Fe_47−x_Mn_28_Co_10_Cr_15_Si_x_ (x: 0 to 10 at.%) showed that there is increase in the T_o_ value with increase in the Si content and reaches to a threshold value of 425 °C for 5 at.% Si. Therefore, it appears that 5 at.% Si would result in effective increase in the metastability of the γ matrix up-to high temperature and also corresponds to the lowering of SFE of the HEA system under consideration.

In order to compare the effect of Si addtion on the γ phase metastability as against the equivalent addition of the Ni or Al in the HEA matrix, T_o_ values and equilibrium γ fractions were obtained for Fe_42_Mn_28_Co_10_Cr_15_Y_5_ (where Y_5_ stands for 5 at.% of elements like Si or Al or Ni) alloys from simulations. It clearly implies that, 5 at.% Si addition results in lowest equilibrium γ fraction (maroon circle in Fig. 1a) and highest T_o_ value as against addition of similar amount of Ni or Al in the alloy (green triangle and blue diamond in Fig. 1a). Thus, Fig. 1a–c prove that 5 at.% Si addition not only makes the Fe-Mn-Cr-Co matrix more metastable but also extend the (γ + ε) phase field to higher temperature in comparison with the Fe-Mn-Cr-Co matrix with or without Ni or Al. Accordingly, the alloy chemistry for the new HEA was fixed to Fe_42_Mn_28_Cr_15_Co_10_Si_5_ (all in at.%).

## Microstructure and Phase Evolution in As-Cast and FSP Conditions

**Figure 2:**
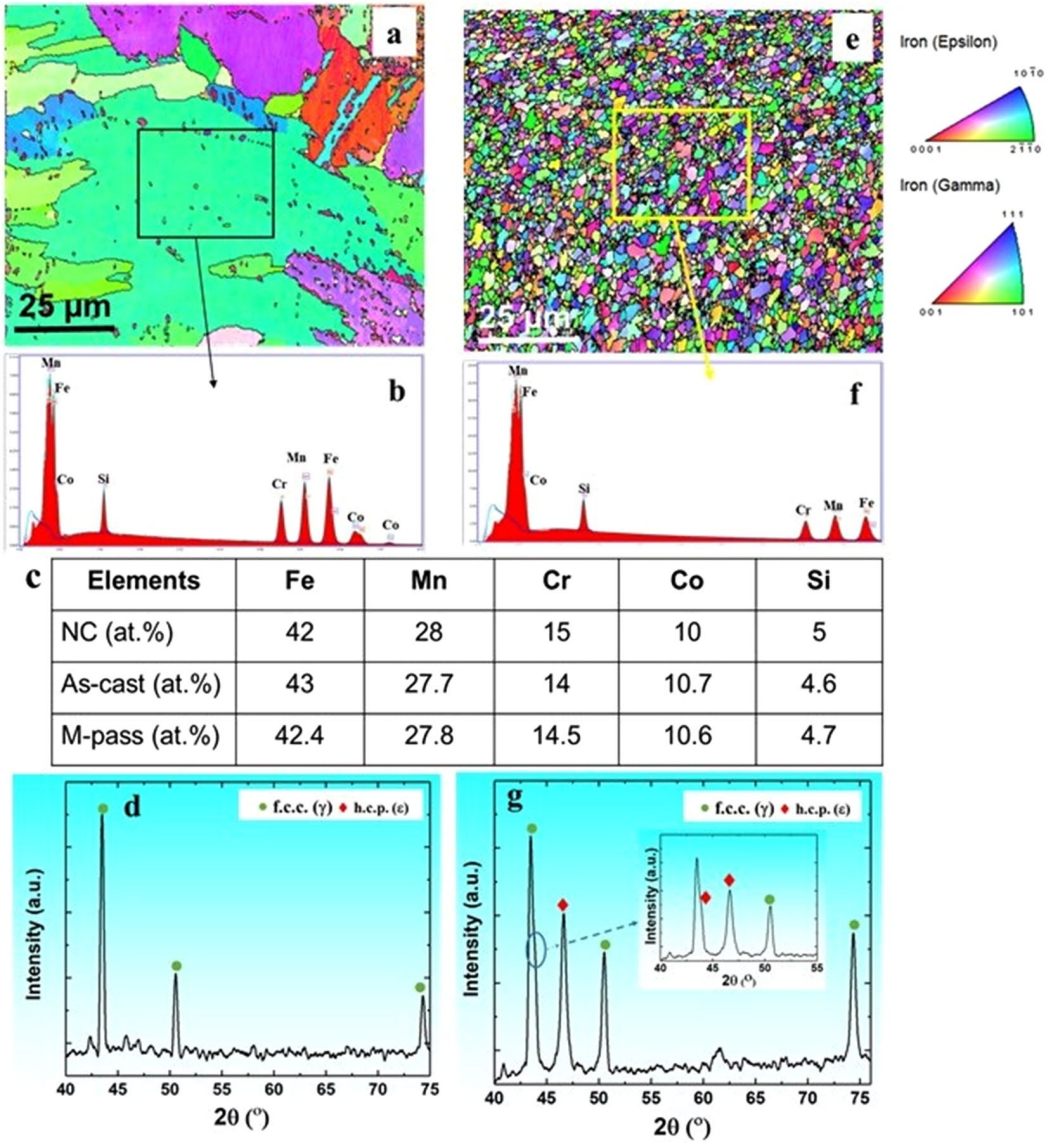
Microstructural characterization of DP-5Si-HEA: (**a**–**d**) EBSD IPF map and corresponding EDS-area spectrum with elemental composition for as-cast DP-5Si-HEA; (**e**–**g**) EBSD IPF map and corresponding EDS-area spectrum with elemental composition for m-pass DP-5Si-HEA. (EBSD: electron back scattered diffraction, IPF: inverse pole figure, EDS: energy dispersive spectroscopy, NC: nominal composition of DP-5Si-HEA).

Figure 2a shows the electron back scattered diffraction (EBSD) inverse pole figure (IPF) map for as-cast Fe_42_Mn_28_Cr_15_Co_10_Si_5_ HEA (hence forth designated as DP-5Si-HEA) along with the corresponding EDS scan and elemental composition (Fig. 2b,c). As expected, addition of Si and Cr resulted in the dual phase microstructure owing to increased metastability of the γ phase^1–5^. As rapid cooling was done during casting, the phase fraction obtained in as-cast material observed to be away from the equilibrium phase fractions. However, presence of distinct peaks for ε phase supports the dual phase nature of the microstructure in DP-5Si-HEA (Fig. 2a and d). The as-cast microstructure was refined by multi-pass friction stir processing (M-pass, FSP carried out with 3 overlapping passes starting with 650 rotations per minute (RPM) down to 350 RPM in the second pass and the last pass being carried out at 250 RPM).

As expected, FSP resulted in refining (grain size (*d)* = 1.3 µm) the as-cast (*d* = 100 µm) microstructure while retaining the chemical homogeneity (Fig. 2c and f) and γ phase dominance (Fig. 2g) in the microstructure. Besides the chemical homogeneity^4,8^, FSP also leads to severe fragmentation of the as-cast dendritic structure into fine equiaxed grains wherein each grain retains different level of strain after processing which resulted in the increase of dislocation storage upon FSP^4,8^. Decrease in the rotational rate during each pass of FSP not only reduces the temperature but also decreases imposed strain during processing and thus limits the dislocation storage. Stored deformation during each pass promotes the conversion of metastable γ phase to ε phase as a result of TRIP. The starting of lower strain rate^8^ processing at 250 RPM during third overlapping pass of FSP limits further nucleation of the ε phase and stabilizes the metastable γ phase in the microstructure at the onset of cooling. Therefore, this kinetic stability of phases triggers the formation of γ phase (88%) dominant microstructure with limited fraction of ε phase (12%) upon M-pass (multi-pass friction stir processing). This was further confirmed by the XRD analysis (Fig. 2g) showing the intense peaks for γ phase along with some distinct peaks for ε phase.

## Improved Strength in As-Cast and FSP DP-5Si-HEA

**Figure 3:**
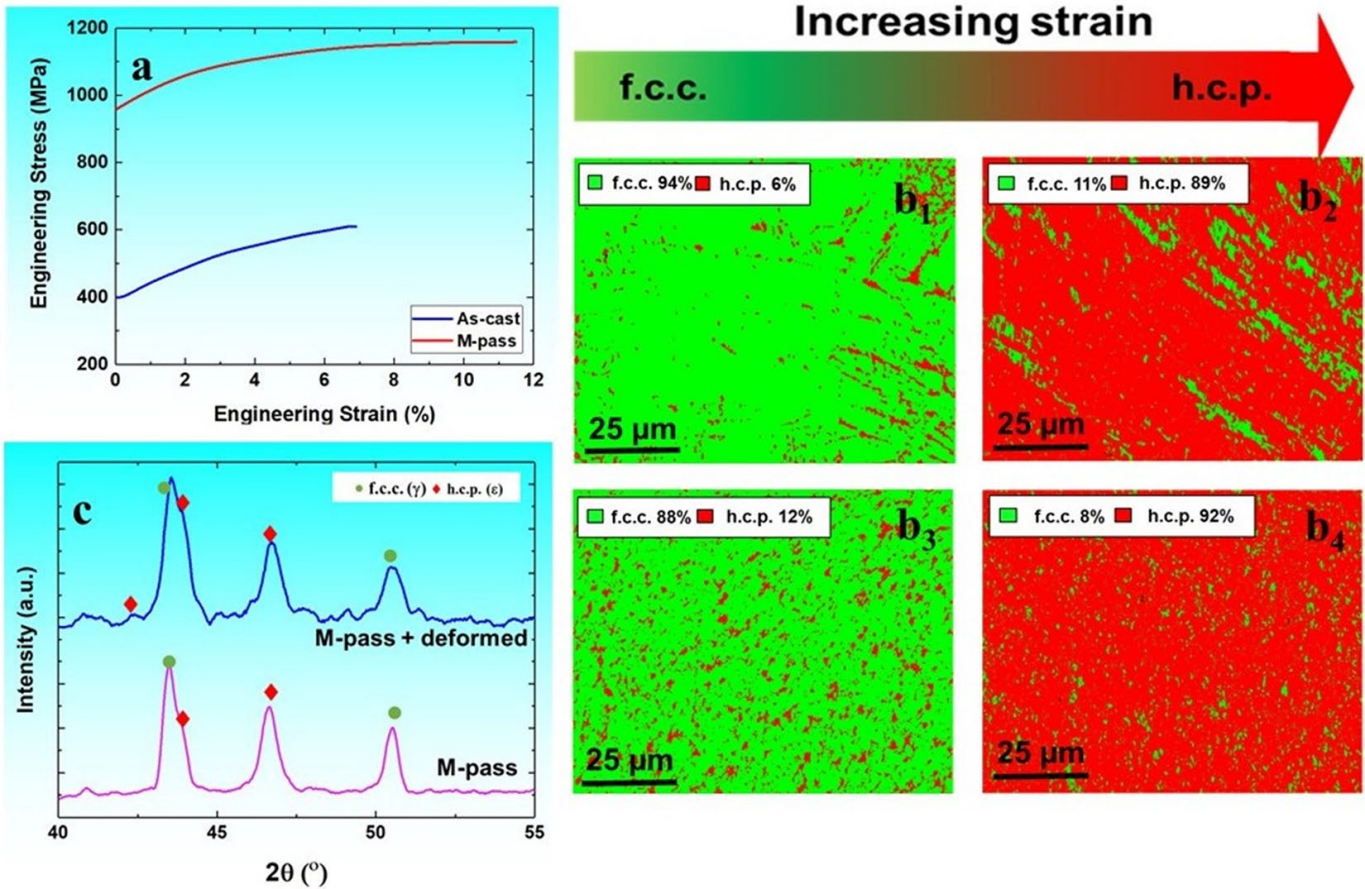
(**a**) Engineering stress-engineering strain curves for DP-5Si-HEA after ambient temperature tensile deformation at 1 × 10^−3^ s^−1^, (**b**_**1**_,**b**_**2**_) EBSD phase map before and after tensile deformation for as-cast condition, and (**b**_**3**_,**b**_**4**_) EBSD phase map before and after tensile deformation for M-pass condition; and (**c**) XRD analysis for M-pass specimen before and after tensile deformation (EBSD: electron back scattered diffraction).

Figure 3a and (b_1_–b_4_) display engineering stress-engineering strain curves and the EBSD phase maps before and after tensile deformation for both as-cast and M-pass specimens respectively. M-pass specimen exhibits very high yield strength (YS) of 950 MPa with a reasonable ductility of 12% in comparison with as-cast material having YS of 400 MPa and ductility of 7%. When compared with the values of YS reported by Li *et al*.^1,2^ for DP-HEA, DP-5Si-HEA showed almost 100 and ~600 MPa increase in YS for as-cast and M-pass specimens respectively.

According to classical work hardening theory^12–15^, increase in dislocation density is an indication of more grain boundary hardening which is also reflected by increased stress for yielding. Thus, the significant increase in the YS of M-pass specimen is mainly attributed to the severe grain refinement from the as-cast grain size of 100 µm to 1.3 µm attained upon FSP. However, along with the grain size, prior ε fraction in the microstructure also plays crucial role in altering the YS of the TRIP alloys^1–4^. This is because, ε phase has different crystal structure than the γ phase and hence ε/γ interfaces act as sites for dislocation pile up thereby increasing the overall dislocation density. As a result, these heavily dislocated martensitic microstructures yield at higher stresses^1–4,16^.

In the present work, M-pass specimen showed very fine grain size and almost two times more ε phase fraction in comparison with the as-cast material. Moreover, the ε phase obtained upon FSP is severely refined and well distributed in the γ matrix as against the as-cast material (Fig. 3b_3_–c). Hence, the onset of yielding was increased in M-pass specimen thereby reaching to a YS of 950 MPa from as-cast YS of 400 MPa. Therefore, the synergistic effect of grain refinement and the phase evolution upon FSP resulted in almost 550 MPa increase in the YS for M-pass specimen in comparison with as-cast condition (Fig. 3a). Also, DP-5Si HEA displayed ultimate tensile strength (UTS) of 1.15 GPa among all DP-HEAs reported so far upon FSP or conventional thermomechanical processing^1–5^. This effective combination UTS and elongation in M-pass specimen is attributed to the higher yet confined work hardening ability in the material which is discussed in upcoming section in detail.

## Rapid Work Hardening and Resultant Mechanical Properties

**Figure 4:**
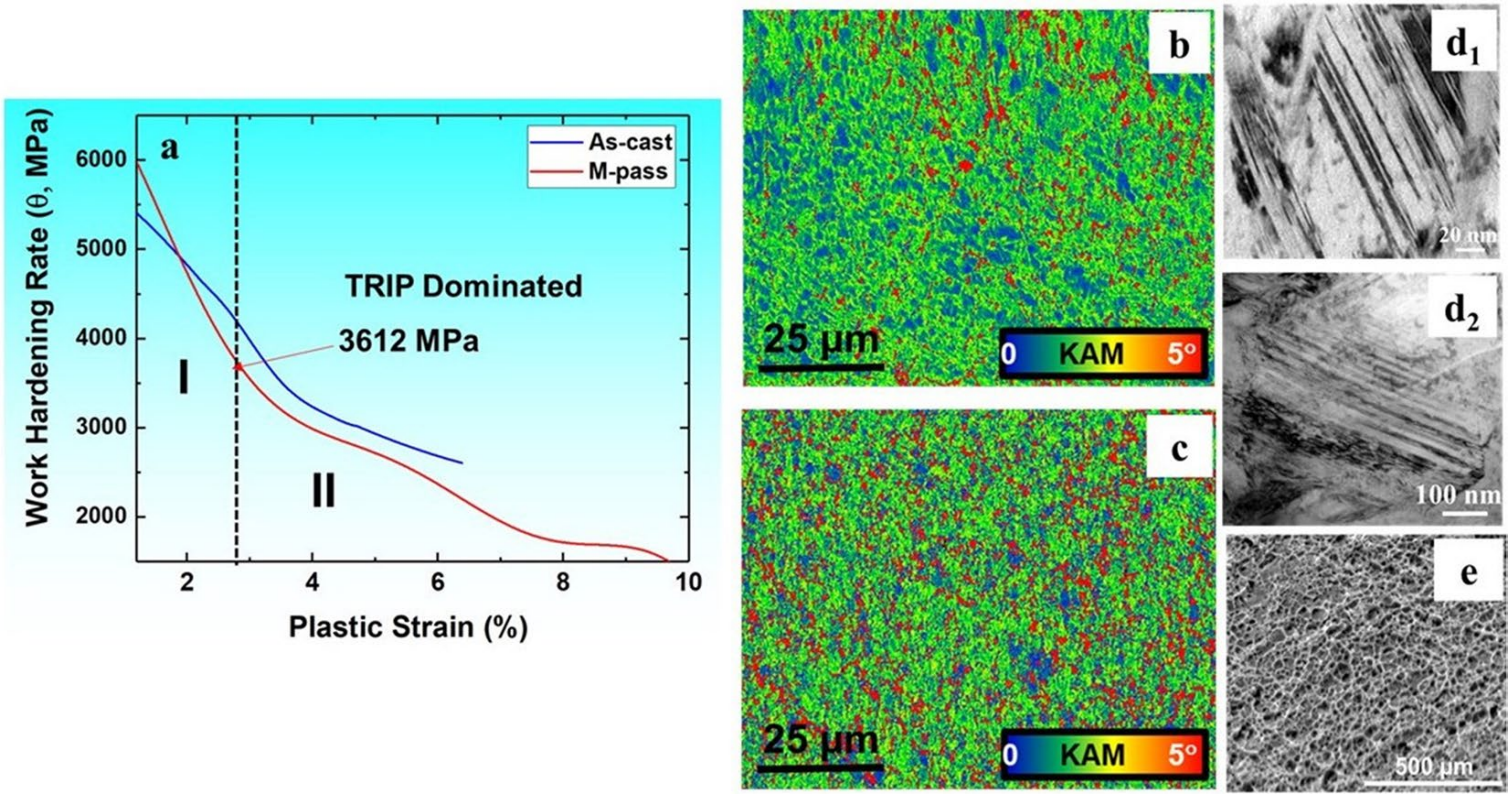
(**a**) Work hardening rate vs plastic strain for as-cast and M-pass conditions; EBSD KAM maps for (**b**) as-cast and (**c**) M-pass specimens, (**d**_**1**_,**d**_**2**_) bright field TEM image of as-deformed M-pass specimen showing presence of deformation twins within h.c.p. (ε) grain, and (**e**) dimpled fracture surface for M-pass condition upon tensile failure. (EBSD: electron back scattered diffraction, KAM: Kernel average misorientation, TEM: transmission electron microscopy).

Figure 4a shows the work hardening (WH) curves for both conditions wherein dominance of stage III during WH is evident, which is expected for the polycrystalline alloys. As can be noted from Figs 3a and 4a, as-cast material itself showed significantly high work hardenability in comparison with conventional steels and newly designed TRIP assisted HEAs^1–4,16^. This is mainly attributed to metastable coarse grained γ enriched starting microstructure which upon subsequent loading can transform rapidly to ε martensite phase as a result of TRIP^17–19^. This transformation effect was captured by the decreased fraction (green color) of γ phase from 94% to 11% in EBSD phase maps (Fig. 3b_1_,b_2_) for as-cast sample after complete tensile deformation.

M-pass condition, however, undergoes rapid drop (steep red curve) in the stage III of WH during the early stages of deformation with sustained WH (θ) value beyond 3612 MPa as marked by the black arrow in Fig. 4. This work hardening value of 3612 MPa corresponds to the onset of change in the slope in the θ vs plastic strain plot in Fig. 4a. This change in slope is due to the predominance of transformation or twinning dominated deformation over dislocation plasticity in material which makes the θ value to be sustained over certain plastic strain^1–4,20–24^. Thus, the WH curve for M-pass specimen gets clearly divided in to two sub-stages of stage III namely dislocation (I) and transformation plasticity dominated (II)^1–4,20–24^. However, rate of work hardening during this sub-stage II (i.e. TRIP) depends on the γ phase stability during deformation for a given grain size and ε phase fraction^1–4^. As reported in our previous work^4^, DP-HEA upon FSP showed almost similar ε phase fraction (~10%) with larger grain size (6.5 µm) in comparison with M-pass specimen in the present work. As a result, DP-HEA^4^ had lower grain boundary area available to inhibit transformation as against the fine grained M-pass specimen during deformation^1–4,17–19^. However, DP-5Si-HEA showed almost 90% change in γ fraction (Fig. 3b_4_) as against 76%^4^ in DP-HEA owing to higher metastable γ phase in former. Thus, the rapid work hardening in the present work is mainly attributed to the higher metastability^20,21^ of the matrix rather than the grain size and prior ε phase fraction. This rapid change in the γ phase fraction (TRIP effect) was also confirmed by XRD analysis (Fig. 3c) showing increased peaks for ε phase after complete tensile deformation.

Moreover, the confined change in the WH rate over plastic strain of 3 to 9% in M-pass condition resulted in reasonable total elongation of 12% as against the as-cast condition. This controlled WH rate in sub-stage II in the WH plot is associated with the uniform strain accommodation by both transforming γ and resultant ε phases^1,3–5^ during deformation in fine grained M-pass specimen which can further be explained by EBSD kernel average misorientation (KAM) maps (Fig. 4b,c) for both the conditions. Strain mismatch between hard ε phase and relatively softer γ phase is evident and needs to be accommodated by geometrically necessary dislocations (GNDs)^1–4,22^. However, more grain boundary area in fine grained M-pass HEA would result in more uniform KAM map (Fig. 4c) relative to the coarse grained HEA (as-cast condition)^1–4^. This is because, higher grain boundary area exerts more back stress and results in more controlled γ → ε transformation and thus sustained work hardening in the very fine grained condition^1–4,22^.

In view of this, coarse grained as-cast material experienced lower plastic strain due to non-uniform strain partitioning among the ε and γ phases (more blue color in ε phase field shown in Fig. 4b) as against in refined M-pass HEA (more green color in ε phase field shown in Fig. 4c). Other than dislocation plasticity, the ε phase can deform by deformation twinning^1–4,18,19^. Figure 4d_1_,d_2_ show the bright field TEM images for the deformed M-pass specimen near the fracture surface which show presence of deformation twins within ε grains (termed as ε twins). ε twin formation in the course of deformation further helps in accommodating strain and impacts the work hardening significantly. Twin boundaries act as stronger barrier to dislocation motion than the dislocation-dislocation interaction. In addition, the formation of twins effectively reduces the spacing between the interfaces resulting in higher flow stresses and altered dislocation storage kinetics at the newly formed twin boundaries^1–4,13–16^. The enhanced interfacial strengthening is referred as the dynamic Hall-Petch effect which promotes more sustained work hardening during deformation and hence results in enhanced plasticity in the material^1–4,13,14^. Moreover, the fine dimple morphology (Fig. 4e) of the fracture surface also confirms the ductile nature of the DP-5Si-HEA upon FSP. In short, controlled interplay between the TRIP in metastable γ phase and the twinning in ε phase for grain refined HEA resulted in almost double elongation (12%) than coarse grained as-cast material. Thus, the ductility of the DP-5Si-HEA can be tailored by engineering the grain size and prior ε phase fraction through appropriate processing route (Fig. 3a).

**Figure 5:**
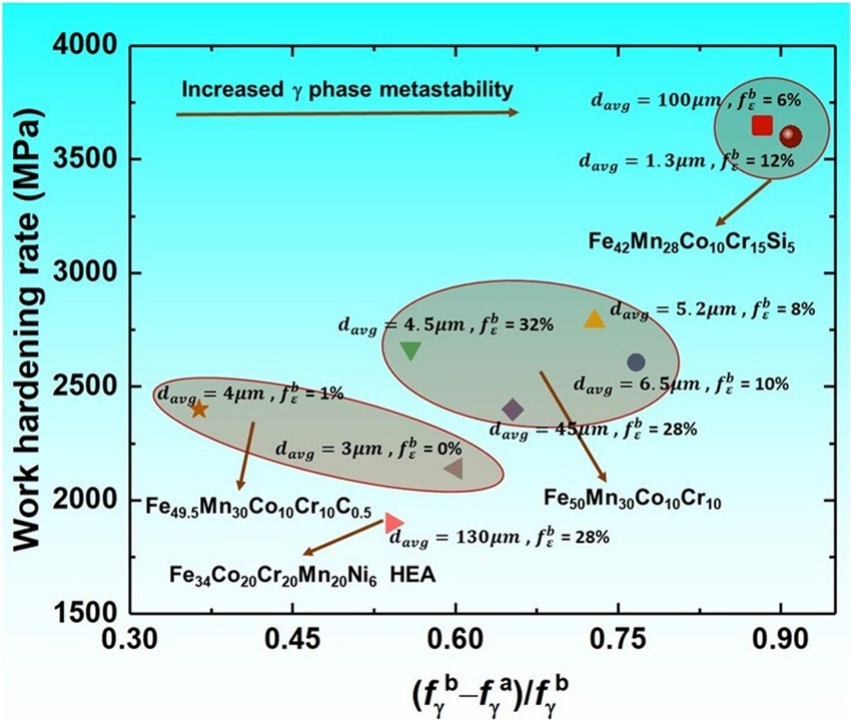
Work hardening rate plotted as a function of fractional change in γ phase after complete tensile deformation for all TRIP assisted HEAs designed till date^1–4,23,25^. (*f*_γ_^b^ = fraction of γ phase before tensile deformation; *f*_γ_^a^ = fraction of γ phase after tensile deformation; *f*_ε_^b^ = fraction of ε phase before tensile deformation; *d*_*avg*_ = average grain size after respective thermomechanical processing).

Figure 5 shows the overall comparison of WH rate values for DP-HEAs with respect to the fractional change in γ phase after complete tensile deformation. The WH rate values used in this plot represents the θ value at the onset of the slope change in the θ vs plastic strain plots obtained from literature on DP-HEAs till date^1–4,23,25^. Moreover, the decrease in the γ fraction upon complete deformation indicates the ability of the material to undergo TRIP over the sustained work hardening regime in the θ vs. plastic strain plot^1–4,20–23^. This is because the change in the γ proportion upon complete deformation for a given grain size and prior ε fraction indicates higher driving force (ΔG^γ→ε^) and hence the preferential tendency for the γ → ε transformation (i.e. γ phase metastability)^5,20^.

Accordingly, fine grained DP-Si5 HEA showed maximum change in the γ fraction irrespective of the grain size and prior ε fraction after complete tensile deformation in comparison with all DP-HEAs reported so far (maroon circle in Fig. 5). Moreover, very coarse grained HEAs^3,23,25^ without Si (light red in Fig. 5) undergone limited change in γ phase fraction as compared to as-cast DP-5Si-HEA (red square in Fig. 5). Hence, among all DP HEAs having different microstructures, Si containing HEAs exhibits highest metastability of γ phase whereas C^3,25^ and Ni^23^ containing DP-HEAs showed lowest. These observations also validate the thermodynamic predictions made in Fig. 1a which showed massive decrease in equilibrium γ fraction for Si containing HEA as against HEAs without Si.

Interestingly, it is also found that the WH rate varies substantially in proportion with the increased metastability of the γ matrix thereby showing almost 1000 MPa rise in the overall WH rate for Si containing HEA. It is also noted that, WH values observed to be similar for HEAs having similar alloy chemistry (Fig. 5). Thus, change in alloy composition (addition of Si increased whereas C and Ni decreased as shown in Fig. 5) predominantly affected the WH rate for DP-HEAs rather than change in the grain size and prior ε fraction upon processing (Fig. 5). As a result, alloy chemistry promoting more metastable γ matrix^20^ undergoes early onset of TRIP and hence display significant increase in UTS and YS. Similar results were obtained in present work wherein DP-5Si-HEA showed highest UTS and YS in comparison with all its counterparts. Hence, tuning the metastability of the γ phase by addition of non-transition elements like Si is a new path way for designing HEAs with higher strength and work hardenability.

## Conclusions

Friction stir processing of Si containing HEA resulted in extremely refined metastable γ phase dominant microstructure thereby exhibiting a very high YS and UTS of 950 MPa and 1.15 GPa, respectively, with an elongation of 12%. These improved mechanical properties in comparison with TRIP assisted HEAs are attributed to the extremely high work hardening ability of the material. This rapid work hardening ability is associated with the increased metastability of γ phase which facilitated the transformation of γ phase and twinning in resultant ε phase during deformation. Therefore, design of metastable DP-5Si-HEA and its microstructural engineering via FSP shows a new pathway for obtaining strong and highly work hardenable HEAs.

## Materials and Methods

The Fe_42_Mn_28_Cr_15_Co_10_Si_5_ HEA (DP-5Si-HEA) was produced by vacuum arc-casting in a cold-copper crucible. The vacuum level achieved was approximately 300 µm, and the chamber was backfilled with argon to 1 atm prior to melt using pure metals with a nominal composition of Fe_42_Mn_28_Cr_15_Co_10_Si_5_ (all in at.%) and ingot dimensions of 250 × 80 × 5 mm^3^. Subsequently, these sheets of 5 mm were subjected to multi-pass friction stir processing (M-pass). M-pass was performed with 3 overlapping passes of FSP with decrement of rotational rates before and after each pass. During each pass of FSP Argon (Ar) gas was flown to shroud the tool-work piece interface for minimizing the exposure to oxygen. The details of the processing parameters used for M-pass are mentioned in Table 1. The processing tool had a shoulder diameter of 12 mm with tapered pin. The root diameter, pin diameter, and length for the tool were 7.5 mm, 6 mm, and 3.5 mm, respectively.

**Table 1:** Processing parameters selected for FSP.

Processing parameters	Pass 1	Pass 2	Pass 3
Rotational Rate (RPM)	650	350	250
Traverse Speed (mm/min)	50.8	50.8	50.8
Plunge Depth (mm)	3.85	3.85	3.85
Tilt Angle (°)	2.0	2.0	2.0

Microstructure of the alloy in as-cast (coarse-grained) and recrystallized (grain-refined) conditions were analyzed by various methods. X-ray diffraction (XRD) measurements were performed on RIGAKU X-Ray equipment using Cu K_α_ radiation operated at 40 kV and 44 mA. Electron backscatter diffraction (EBSD) measurements were carried out by a FEI NOVA Nano (SEM) with a Hikari camera and the TSL OIM 8 data collection software. Rectangular 1 mm-thick, dog-bone-shaped mini-tensile specimens were machined using a mini computer numerical control (CNC) machine from 1 mm below from the surface within the nugget region of the FSP specimen whereas from the top surface of the as-cast ingot. Gage length and width of the tensile specimens were 5 and 1.25 mm, respectively. In each condition, three samples were tested at room temperature at an initial strain rate of 10^−3^ s^−1^ to confirm reproducibility of the results.

## References

[1] Li Z (2016). Metastable high-entropy dual-phase alloys overcome the strength-ductility trade-off. Nature.

[2] Li Z (2017). A TRIP-assisted dual-phase high-entropy alloy: grain size and phase fraction effects on deformation behavior. Acta Mater..

[3] Li Z (2017). Interstitial atoms enable joint twinning and transformation induced plasticity in strong and ductile high-entropy alloys. Sci. Reports..

[4] Nene SS (2017). Enhanced strengh and ductility in friction stir processed engineered high entropy alloy. Sci. Rep..

[5] Xiong R (2014). Thermodynamic calculation of stacking fault energy of the Fe-Mn-Si-C high manganese steels. Mater. Sci. Eng. A.

[6] Pierce DT (2014). The influence of manganese content on the stacking fault and austenite/ε -martensite interfacial energies in Fe – Mn –(Al – Si) steels investigated by experiment and theory. Acta Mater..

[7] Li Z, Raabe D (2017). Strong and ductile non-equiatomic high-entropy alloys: design, processing, microstructure, and mechanical properties. JOM..

[8] Palanivel S (2016). A framework for shear driven dissolution of thermally stable particles during friction stir welding and processing. Mater. Sci. Eng. A.

[9] Galindo-Nava EI, Rivera-Díaz-del-Castillo PEJ (2017). Understanding martensite and twin formation in austenitic steels: A model describing TRIP and TWIP effects. Acta Mater..

[10] Pustov LY (2008). Face-centered cubic phase stability and martensitic transformation under deformation in Fe–Ni and Fe–Mn alloys nanostructured by mechanical alloying and high-pressure torsion. Mater. Sci. Eng., A.

[11] Pisarik ST, Van Aken DC (2016). Thermodynamic driving force of the γ → ε transformation and resulting M_S_ temperature in high-Mn steels. Metall. Mater. Trans. A..

[12] Yeh J (2004). Nanostructured high entropy alloys with multiple component elements: novel alloy design concepts and outcomes. Adv. Eng. Mater..

[13] Cooman DB, Estrin Y, Kim S (2018). Twinning induced plasticity steels. Acta. Mater..

[14] Cordero ZC, Knight BE, Schuh CA (2015). Six decades of the Hall-Petch effect-a survey of grain size strenthening studies on pure metals. Intl. Mater. Rev..

[15] Song R, Ponge D, Raabe D (2005). Improvement of the work hardening rate of ultrafine grained steels through second phase particles. Scr. Mater..

[16] He BB (2017). High dislocation density–induced large ductility in deformed and partitioned steels. Science.

[17] Trichter F (1978). A study γ → ε phase transformation in Fe-Mn alloys induced by high pressure and plastic deformation. Scripta Metall.

[18] Martin S, Ullrich C, Rafaja D (2015). Deformation of austenitic CrMnNi TRIP/TWIP Steels: nature and role of the ε martensite. Mater. Today Proc..

[19] Herrera C, Ponge D, Raabe D (2011). Design of a novel Mn-based 1 GPa duplex stainless TRIP steel with 60% ductility by a reduction of austenite stability. Acta Mater..

[20] Papula S (2016). Strain hardening of cold-rolled lean-alloyed metastable ferritic-austenitic stainless steels. Mater. Sci. Eng. A.

[21] Pierce DT (2015). The influence of stacking fault energy on the microstructural and strain- hardening evolution of Fe – Mn – Al – Si steels during tensile deformation. Acta Mater..

[22] Calcagnotto M (2010). Orientation gradients and geometrically necessary dislocations in ultrafine grained dual-phase steels studied by 2D and 3D EBSD. Mater. Sci. Eng. A.

[23] Li Z (2017). Ab initio assisted design of quinary dual-phase high-entropy alloys with transformation-induced plasticity. Acta Mater..

[24] Kumar N (2015). Friction stir processing of a high entropy alloy Al_0.1_CoCrFeNi. JOM..

[25] Frank, M. *et al*. Dynamic phase stabilization and resultant mechanical propeties upon friction stir processing of a metstable interstitial high entropy alloy, Manuscript to be submitted (2018).

